# Research on the osteogenesis and biosafety of ECM–Loaded 3D–Printed Gel/SA/58sBG scaffolds

**DOI:** 10.3389/fbioe.2022.973886

**Published:** 2022-08-17

**Authors:** Guozhong Tan, Rongfeng Chen, Xinran Tu, Liyang Guo, Lvhua Guo, Jingyi Xu, Chengfei Zhang, Ting Zou, Shuyu Sun, Qianzhou Jiang

**Affiliations:** ^1^ Department of Endodontics, Affiliated Stomatology Hospital of Guangzhou Medical University, Guangdong Engineering Research Center of Oral Restoration and Reconstruction, Guangzhou Key Laboratory of Basic and Applied Research of Oral Regenerative Medicine, Guangzhou, China; ^2^ Department of Oral and Maxillofacial Surgery, Affiliated Stomatology Hospital of Guangzhou Medical University, Guangdong Engineering Research Center of Oral Restoration and Reconstruction, Guangzhou Key Laboratory of Basic and Applied Research of Oral Regenerative Medicine, Guangzhou, China; ^3^ Endodontology, Restorative Dental Sciences, Faculty of Dentistry, The University of Hong Kong, Hong Kong, China; ^4^ Department of Endodontics, Stomatological Hospital, Southern Medical University, Guangzhou, China

**Keywords:** 3D printing, ECM, scaffolds, bone defects, biosafety, bone regeneration

## Abstract

Employing scaffolds containing cell–derived extracellular matrix (ECM) as an alternative strategy for the regeneration of bone defects has shown prominent advantages. Here, gelatin (Gel), sodium alginate (SA) and 58s bioactive glass (58sBG) were incorporated into deionized water to form ink, which was further fabricated into composite scaffolds by the 3D printing technique. Then, rat aortic endothelial cells (RAOECs) or rat bone mesenchymal stem cells (RBMSCs) were seeded on the scaffolds. After decellularization, two kinds of ECM–loaded scaffolds (RAOECs–ECM scaffold and RBMSCs–ECM scaffold) were obtained. The morphological characteristics of the scaffolds were assessed meticulously by scanning electron microscopy (SEM). In addition, the effects of scaffolds on the proliferation, adhesion, and osteogenic and angiogenic differentiation of RBMSCs were evaluated by Calcein AM staining and reverse transcription polymerase chain reaction (RT–PCR). *In vivo*, full–thickness bone defects with a diameter of 5 mm were made in the mandibles of Sprague–Dawley (SD) rats to assess the bone regeneration ability and biosafety of the scaffolds at 4, 8 and 16 weeks. The osteogenic and angiogenic potential of the scaffolds were investigated by microcomputed tomography (Micro–CT) and histological analysis. The biosafety of the scaffolds was evaluated by blood biochemical indices and histological staining of the liver, kidney and cerebrum. The results showed that the ECM–loaded scaffolds were successfully prepared, exhibiting interconnected pores and a gel–like ECM distributed on their surfaces. Consistently, *in vitro* experiments demonstrated that the scaffolds displayed favourable cytocompatibility. *In vitro* osteogenic differentiation studies showed that scaffolds coated with ECM could significantly increase the expression of osteogenic and angiogenic genes. In addition, the results from mandibular defect repair *in vivo* revealed that the ECM–loaded scaffolds effectively promoted the healing of bone defects when compared to the pure scaffold. Overall, these findings demonstrate that both RAOECs–ECM scaffold and RBMSCs–ECM scaffold can greatly enhance bone formation with good biocompatibility and thus have potential for clinical application in bone regeneration.

## 1 Introduction

Bone defects of the oral and maxillofacial areas derived from congenital defects, trauma, infection or surgical removal usually need surgical external intervention to enhance regeneration. Autografts are considered the gold standard in bone repair but can cause donor site morbidity, and the sources are limited (Agarwal and García Andrés, 2015). Allografts and xenografts can also have osteoconductive and osteoinductive properties but suffer from the risks of immune rejection and pathogen transmission (Lee et al., 2019; Zhu et al., 2020). As a result, synthetic bone substitutes have been the focus of attention and not only can be produced on a large scale but also possess osteogenic properties. In our previous study, gelatin (Gel), sodium alginate (SA) and bioactive glass (BG) were deemed to have promising prospects for fabricating composite scaffolds by 3D printing technology, which showed that the prepared scaffolds have good osteogenic induction performance (Wu J. et al., 2019).

To improve the osteogenic potential of engineering scaffolds, bioactive factors with osteoinductive activity are integrated into the scaffolds to provide guidance for cell differentiation or tissue regeneration. The extracellular matrix (ECM), a complex network with a noncellular component, is composed of various structural and functional molecules secreted by cells, such as collagen, fibronectin, laminin, glycosaminoglycans, and proteoglycans (Carvalho Marta et al., 2019). The functional molecules of the ECM can produce a native microenvironment to improve cell proliferation, adhesion and differentiation. Several studies have attempted to imitate the ECM microenvironment by integrating supporting molecules into synthetic biomaterials, but they provide limited components that present specific functional receptors for cell attachment or proliferation and cannot satisfy the entire function of native ECM (Wu Y.-H. A. et al., 2019b). Natural ECM, with its complex structure and composition, is difficult to synthesize artificially. Moreover, it is hard to realize application by using a single ECM as the mechanical properties of ECM are poor; therefore, the combined application of scaffolds and ECM is currently a popular approach. Studies are aimed at incorporating natural ECM from specific tissue or cultured cells into scaffolds, which produces cell–or tissue–specific cues to enhance osteogenesis and has been receiving growing research attention. In particularly, cell–derived ECM can be easily obtained through cell culture, proliferation and differentiation, followed by decellularization, which can be combined with scaffolds to enhance the osteogenic ability and has been reported by numerous studies (Pati et al., 2015; Kim et al., 2018).

Inspired by this, we attempted to ornament the Gel/SA/58sBG scaffolds with cell–derived ECM to induce angiogenesis and accelerate bone repair. Adequate blood supply is also crucial for bone regeneration of scaffolds after implantation, which involves the interaction of a series of precursor cells, growth factors and angiogenic factors. The lack of vasculature in tissue engineered scaffolds could result in inadequate oxygen and nutrition supply and waste removal, eventually leading to hypoxia and cell death. To our knowledge, one of the most effective strategies to improve the angiogenic and osteogenic potential of scaffolds is to combine endothelial progenitor cells (EPCs)–derived ECM (Peng et al., 2019; Lin et al., 2021). EPCs are the precursor of endothelial cells, and transplanted EPCs reportedly stimulate angiogenesis by differentiating into mature endothelial cells or triggering angiogenic events by secreting various trophic factors (Ishikawa and Asahara, 2004; Critser and Yoder, 2010; Kim et al., 2010; Ackermann et al., 2014). Rat aortic endothelial cells (RAOECs) are types of endothelial cells that are commonly used for research on cardiovascular diseases. Recently, RAOECs have also been used to study fracture healing and bone tissue engineering (Pekozer Gorke et al., 2016). Considering that the extracellular matrix plays a vital role in angiogenesis, we hypothesize that RAOECs–derived ECM combined with scaffolds may produce an effective way to induce angiogenesis. Bone marrow mesenchymal stem cells (BMSCs) with superior osteoconductive potential are a primary choice in bone tissue engineering applications, as they promote vascularization through paracrine action and differentiate into osteoblasts and are thus beneficial for bone regeneration and angiogenesis (Chen et al., 2018; Ying et al., 2020). Several studies have investigated the abilities of BMSCs–derived ECM to induce bone tissue regeneration (Wei et al., 2015; Gao et al., 2018; Sun et al., 2018; Chi et al., 2020). For our research, 3D–printed Gel/SA/58sBG scaffolds exhibit several advantages, including porous structure and good mechanical characteristics, which also contribute to oxygenation up-take, waste excretion and effective support. With the appropriate pore size and porosity, the cell–derived ECM can enter and adhere to the scaffolds to promote angiogenesis and osteogenesis. An appealing option is to combine RBMSCs–or RAOECs–derived ECM with scaffolds to simulate the natural osteogenic bone microenvironment for bone repairing. Therefore, we seeded RBMSCs or RAOECs on Gel/SA/58sBG scaffolds to obtain two kinds of cell–derived ECM scaffolds by decellularized treatment, which meet all the characteristics of functional scaffolds, including simulating the complex composition of the cell–derived ECM and the interactions among various macromolecules *in vitro* and *vivo*. The aim of this study was to investigate the osteogenic potential and biosafety of the two kinds of cell–derived ECM scaffolds.

In this research, Gel, SA and 58sBG were mixed according to a certain proportion to form ink, and scaffolds were fabricated by 3D printing. The scaffolds were cultured with RAOECs or RBMSCs and loaded with ECM after decellularization. *In vitro*, we assessed the surface morphology, cytocompatibility and osteogenic differentiation of the scaffolds. The bone regeneration capability and biosafety of the scaffolds *in vivo* were evaluated after implantation into mandibular bone defects of rats. Ultimately, both kinds of ECM–loaded scaffolds were proven to be more suitable for bone regeneration with favourable cytocompatibility, osteogenic activity and biosafety.

## 2 Materials and methods

### 2.1 Fabrication of 3D–printed Gel/SA/58s BG scaffolds

58sBG with a composition of TEOS (6.6 ml), TEP (0.86 ml) and Ca(NO_3_)_2_ 4H_2_O (4.25 g) was prepared by an evaporation–induced self–assembly (EISA) method as previously reported (Tsigkou et al., 2014). The ink was first prepared before printing. A total of 1.05 g 58sBG, 1.5 g Gel and 0.6 g of SA were added to 10 ml of deionized water at 55°C in a constant temperature system. Then, magnetic and mechanical stirring were used to mix the materials. The obtained ink was transferred to the barrel of a 3D bioprinter (Regenovo, Hangzhou, China). The experimental parameters of the printing process were set as follows: the needle diameter was 0.4 mm, the extrusion pressure was 0.38 MPa, the printing speed was 15 mm/s, the adjacent filaments were 1.2 mm, and the extrusion temperature was 28°C. According to the needs, two different shapes of scaffolds were printed (10 mm × 10 mm × 1.5 mm cubic scaffold for *in vitro* study, 5 mm diameter and 1.5 mm high cylindrical scaffold for *in vivo* study). Next, the obtained scaffolds were soaked in 10% CaCl_2_ solution and cross–linked for 10 min. Further cross–linking in 0.25% glutaraldehyde solution for 30 min was carried out. After that, scaffolds were washed with distilled water 5 times and soaked in distilled water for 8 h. Finally, scaffolds were frozen at 80°C and dried for 24 h in a freeze dryer (CHRIST, Germany) for further use.

### 2.2 Cell culture

RAOECs, RBMSCs (Procell, Wuhan, China), and L929 (iCell Bioscience Inc., Shanghai, China) cells were cultured with Dulbecco’s modified Eagle’s medium (DMEM) supplemented with 10% foetal bovine serum (Gibco, Thermo Fisher Scientific, Inc., United States). The cells were maintained at 37°C in a humidified atmosphere with 5% CO_2_. These cells were then subcultured and frozen for storage for later use.

### 2.3 Preparation of ECM loaded–3D–printed Gel/SA/58s BG scaffolds

Before seeding, all cells were expanded in standard culture medium in a monolayer until they reached 80%–90% confluence. RAOECs or RBMSCs were seeded on scaffolds, which were placed in 6–well plates at passages 3–5 with a density of 2 × 10^5^ cells per scaffold. The medium was changed every 2 days. After 14 days of cultivation and proliferation, the scaffolds were transferred to new well plates to remove nonadherent cells. Based on previously reported methods (Kang et al., 2012; Gong et al., 2017), the cell–scaffold composites were immersed in 0.1% ammonium hydroxide (NH_4_OH) + 0.5% Triton X–100 (Sigma–Aldrich, St. Louis, MO) solution for 30 min and then washed with PBS 3 times. All vital cells were removed for decellularization, and RAOECs–ECM scaffold and RBMSCs–ECM scaffold were obtained. Finally, the ECM–loaded scaffolds were frozen for further use.

### 2.4 Cell attachment and morphology

To detect the cell viability and attachment of the RBMSCs seeded on the pure scaffold, RAOECs–ECM scaffold or RBMSCs–ECM scaffold. RBMSCs (4 ×10^5^ cells/scaffold) were seeded onto the scaffolds and incubated at 37°C. After 1, 3 and 7 days, the medium was removed, and the scaffolds were washed with PBS three times and then treated with Calcein AM (Bestbio, Shanghai, China). The live cells on the scaffolds were observed under a fluorescence microscope. To observe the attachment and morphology of cells grown on the scaffolds, the cell–scaffold composites were collected at Day 3, washed twice with PBS and chemically fixed using 2.5% glutaraldehyde solution. The specimens were subsequently dehydrated twice with a series of graded ethanol (30%, 50%, 70%, 80%, 90%, and 100%). After dehydration, the scaffolds were immersed in hexamethyl–disilazane for 2 min and vacuum–dried overnight. Finally, the scaffolds were sputter–coated for 60 s at 10 mA with gold, and the gold–coated scaffolds were observed via SEM.

### 2.5 Gene expression by real–time polymerase chain reaction

After RBMSCs were seeded on the scaffolds for 7 and 14 days, the scaffolds were removed from the medium and washed twice with PBS. Total RNA from each group was extracted using an RNA extraction kit (AG21017, Accurate Biology, China) and quantified by a spectrophotometer (NanoDrop 2000; Thermo Fisher Scientific, Waltham, MA, United States). The obtained RNA was used to synthesize complementary DNA (cDNA) *via* an RT–PCR Kit (AG11706, Accurate Biology, China). Finally, RT–PCR was performed using SYBR^®^ Premix Ex TaqTM II (RR820A, Takara, Japan) in a CFX96 Real–time PCR machine (Bio–Rad, Hercules, CA, United States). The relative gene expression was calculated using 2^−∆∆Ct^. GAPDH was used as the housekeeping gene, and the genes examined were RUNX2, BMP2, CD31 and VEGF. The primer sequences are detailed in Table 1.

**TABLE 1:** RT–PCR primers.

Gene/primer target	Forward primer (5′–>3′)	Reverse primer (5′–>3′)
GAPDH	TCT​CTG​CTC​CTC​CCT​GTT​C	ACA​CCG​ACC​TTC​ACC​ATC​T
RUNX2	GAA​ATG​CCT​CTG​CTG​TTA​TGA	AAG​TGA​AAC​TCT​TGC​CTC​GTC
BMP2	GAG​AAA​AGC​GTC​AAG​CCA​AAC	GTC​ATT​CCA​CCC​CAC​ATC​ACT
CD31	AGA​ATC​CGC​CCT​GGA​GTG​TT	ACG​GCA​GCA​GAG​CAG​AAA​GC
VEGF	CCA​CGA​CAG​AAG​GGG​AGC​A	ACA​CCG​CAT​TAG​GGG​CAC​A

### 2.6 Preparation of mandibular bone defect

A total of 108 male SD rats weighing 280–320 g were purchased from the experimental animal centre of Guangzhou University of Chinese Medicine (licence number: SCXK (Guangdong) 2018–0034) and divided into four groups (*n* = 9 each group at each time point): 1) control group; 2) scaffold group; 3) RAOECs–ECM scaffold group; and 4) RBMSCs–ECM scaffold group. Briefly, general anaesthesia was achieved by intraperitoneally injecting pentobarbital (40 mg/kg), and a 15 mm longitudinal incision was made 2 mm above the lower edge of the mandible body to expose the bone surface when adequacy of the anaesthesia had been confirmed. Then, 5 mm full–thickness critical defects were created on the right side of the mandibular ramus of the rats by using a 5 mm trephine bur. Physiological saline irrigation was maintained throughout the drilling procedure to prevent heat damage. The fillings of the defects were randomly assigned. Defects were filled with pure scaffold, RAOECs–ECM scaffold or RBMSCs–ECM scaffold, respectively. The control group was not filled with any material. The wounds were closed with sutures in layers, and penicillin sodium (160,000 IU/ml) was intramuscularly injected in the first 3 days after surgery. Rats were euthanized via carbon dioxide asphyxiation at 4, 8 and 16 weeks postsurgery. The mandibles were harvested and soaked in 4% paraformaldehyde for further analysis.

### 2.7 Microcomputed tomography (Micro–CT) test

To analyse the newly formed bone tissue in the defect area, five mandibles of each group were scanned by a Micro–CT scanner (SkyScan 1,174 Compact Micro CT, Kontich, Belgium) with a resolution of 10 μm at each time point. Mandibles were dried with a paper towel to remove the residual paraformaldehyde solution and perpendicularly stabilized in the exact centre of the 4 cm diameter cylindrical moulds. After that, Micro–CT scans were performed, and transverse sections were acquired from the raw data processed by NRecon software. The obtained sections were transferred to CTan software for quantitative analysis, which included the parameters of total volume (TV, mm^3^), bone volume (BV, mm^3^), percent of bone volume (BV/TV, %) and bone mineral density (BMD, mg/cm^3^).

### 2.8 Microfil perfusion

After full anaesthesia, four rats from each group at each time point were supine on the operating table and cut from the sternal notch to the midline of the abdomen. Then, the thorax and pericardium were cut open with scissors to expose the heart. The left ventricle was inserted with a blunt 18–size needle, and 300 ml of heparin normal saline and 4% paraformaldehyde were injected successively. Then, 18 ml of Microfil (Microfil MV–120, Flow Tech) was perfused. When the blood vessel was infused with the contrast agent, staining of the tongue and the coronary artery was visible. The mandibular bones of the carcasses were removed and stored overnight in a refrigerator at 4°C, kept in 4% paraformaldehyde for 24 h, and fully decalcified in 10% ethylene diamine tetraacetic acid (EDTA) solution for approximately 2 months. Micro–CT was used to scan the mandibular defects after decalcification, and neovascularization was detected by 3D reconstruction imaging. The area of local blood vessels in bone defects was evaluated by CTvox software.

### 2.9 Histological and immunohistological analyses

After incubation in 4% paraformaldehyde for 24 h and decalcification in 10% EDTA for 2 months, the obtained samples were dehydrated with alcohol gradients and embedded in paraffin. Then, the samples were cut longitudinally along the axial plane to make 4–μm–thick sections. The sections from each sample were subjected to haematoxylin–eosin (H&E) and Masson’s trichrome (MT) staining. Histologic observations and images were acquired by light microscopy under ×10 and ×200 magnification. To evaluate the osteogenesis and angiogenesis of the defects, immunohistochemical (IHC) staining of RUNX2, OCN, CD31 and VEGF was performed. Rabbit anti–RUNX2 (1:50, Abcam, United Kingdom), rabbit anti–OCN antibody (1:200, Bioss, United States), rabbit anti–CD31 antibody (1:1,000, Abcam, United Kingdom) and rabbit anti–VEGF antibody (1:200, Bioss, United States) were used as the primary antibodies. After dewaxing and rehydration, antigen retrieval was performed in a pressure cooker for 10 min. Subsequently, 3% hydrogen peroxide (Boster, China) and 10% bovine serum albumin (Solarbio, China) were used to eliminate endogenous peroxidase activity and block nonspecific antibody binding sites, respectively. The sections were then incubated with the primary antibodies overnight at 4°C. Next, the sections were incubated with the secondary antibodies (Beyotime Biotechnology, China) against rabbit IgG, and diaminobenzidine (DAB) (Cell Signaling Technology, United States) was used to show the positive staining. Finally, the sections were counterstained with haematoxylin, and images were captured with a light microscope (Leica, Germany). For quantitative analysis, the percentage of the new bone area in H&E staining was calculated with the new bone area/tissue area × 100%. For IHC analysis, the average optical density (AOD) was quantitatively measured by ImageJ.

### 2.10 Histopathological and biochemical analyses

At each time point after surgery, 5 rats from each group were fixed in a metabolism cage for blood collection. In brief, a 2 ml blood sample from each rat was drawn at each time point via the caudal vein, which was used to analyse the levels of albumin (ALB), total protein (TP), aspartate aminotransferase (AST), alanine transaminase (ALT), creatinine (Cr) and blood urea nitrogen (BUN) in the Guangzhou Key Laboratory of Basic and Applied Research of Oral Regenerative Medicine. After that, animals were sacrificed via carbon dioxide asphyxiation. The liver, kidney and cerebrum were collected from rats, which were fixed with 4% paraformaldehyde for 24 h, embedded in paraffin, and stained with haematoxylin–eosin (H&E) at a 4 μm thickness to reveal if pathological changes had occurred.

### 2.11 Statistical analysis

All data are presented as the mean ± standard deviation for *n* ≥ 3. SPSS (IBM SPSS Inc., United States) demonstrated that all data were in accordance with a normal distribution. Statistical significance between groups was tested using one–way analysis of variance (ANOVA) followed by Tukey’s post–hoc test. *p* < 0.05 was considered statistically significant.

## 3 Results

### 3.1 Morphology and characterization of scaffolds

**FIGURE 1:**
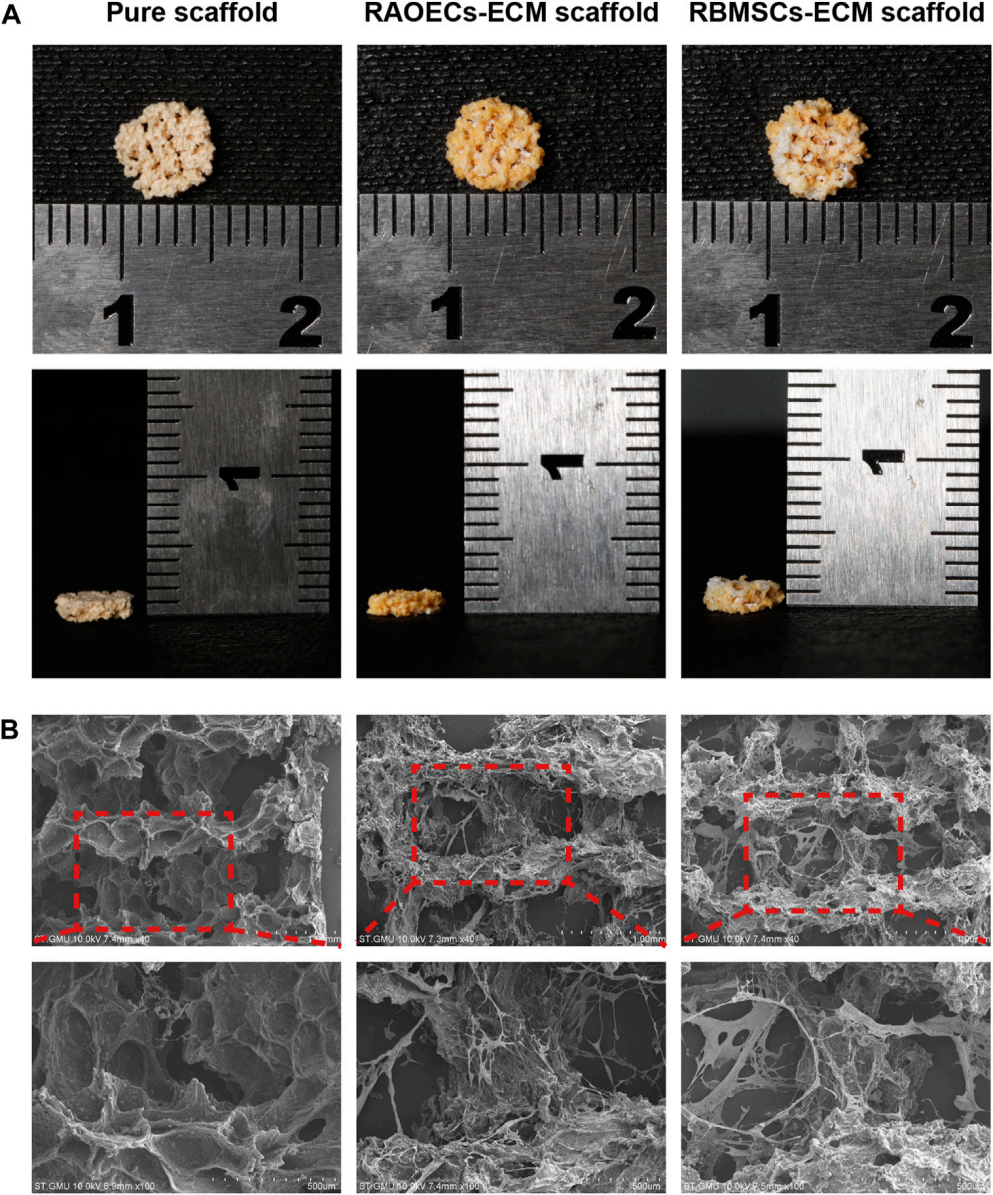
Morphological characteristics of the pure scaffold, RAOECs–ECM scaffold and RBMSCs–ECM scaffold. **(A)** Optical images of fabricated scaffolds with cylindrical shapes were observed from the top view and side view. **(B)** SEM images at various magnifications showed the surface morphology and microstructure of the scaffolds.

The general morphology and surface characterization of the scaffolds are shown in Figure 1 **(A)** The general morphology of the scaffolds was observed. **(B)** The surface morphologies and internal structures of the freeze–dried scaffolds were evaluated by SEM. According to different needs, we prepared scaffolds with cylindrical shapes (diameter: 5 mm, height: 5 mm), which were applied for the experiments *in vivo*. All the scaffolds showed a complete macrostructure with multiple pores, rough walls and connected apertures, which is conducive to cell recruitment, adhesion and differentiation. The addition of ECM indicated that the scaffold surface was covered with a biological layer, generating a more homogenous porous structure, which further increased cellular infiltration.

### 3.2 Cell adhesion, proliferation and RT–PCR assay

**FIGURE 2:**
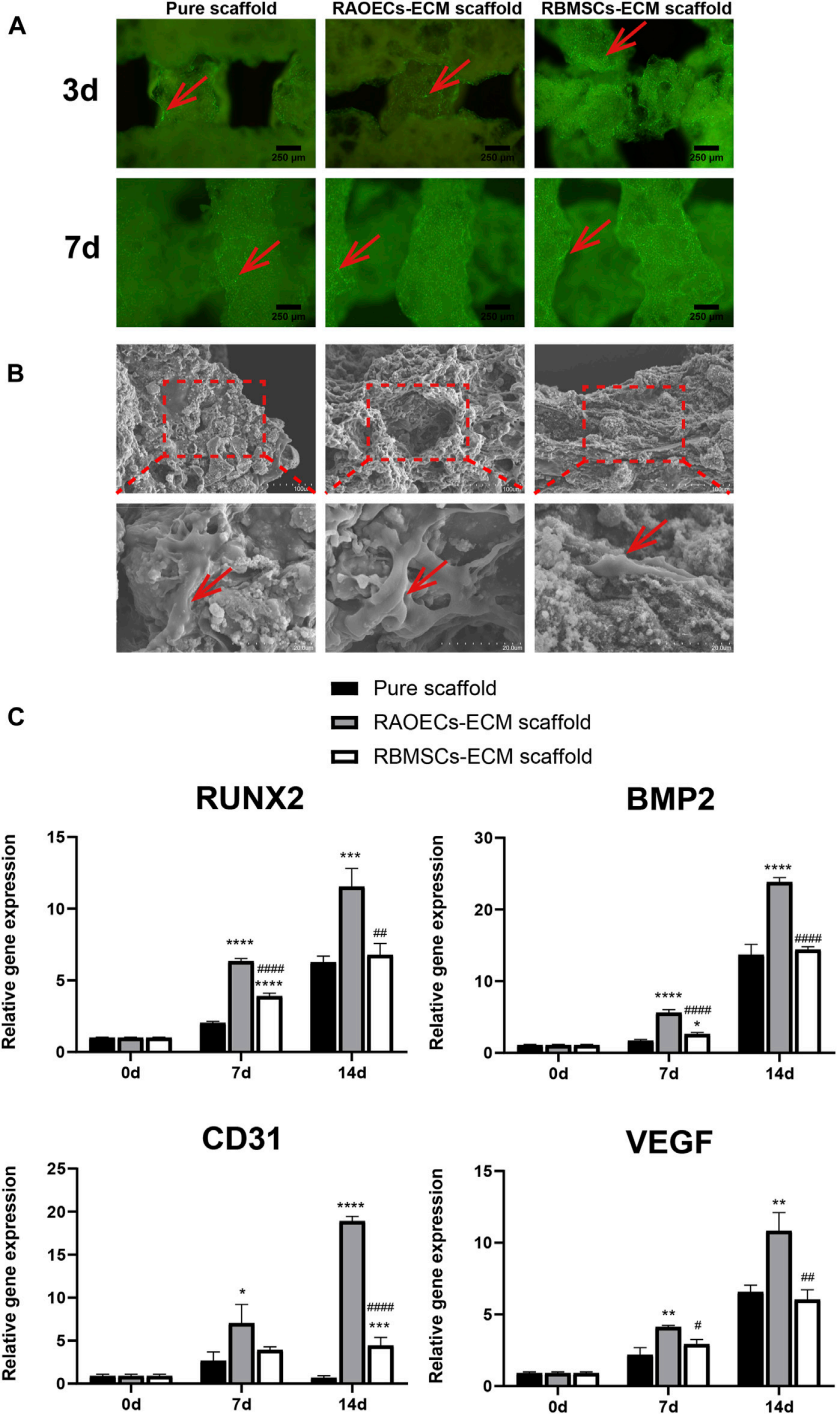
Cell adhesion, proliferation and RT–PCR assessment. **(A)** Fluorescence microscopy images of RBMSCs–seeded scaffolds after Calcein AM staining. **(B)** Scanning electron micrographs of RBMSCs (arrows) adhering to the surfaces of scaffolds at various magnifications. **(C)** The expression levels of osteogenic and vascularization–related genes after 0, 7 and 14 d of induction. (Significant effect compared to pure scaffold: ∗*p* < 0.05, ∗∗*p* < 0.01, ∗∗∗*p* < 0.001, ∗∗∗∗*p* < 0.0001. Significant effect compared to the RAOECs–ECM scaffold: #*p* < 0.05, ##*p* < 0.01, ####*p* < 0.0001, *n* = 3.)

As shown in Figure 2A, Calcein AM staining confirmed the occurrence of viable cells on the porous structures of scaffolds, and the homogeneous distribution of viable cells was more evident at ECM–loaded scaffolds, which demonstrated that ECM provides an appropriate microenvironment and a biocompatible surface for RBMSCs adhesion. (Figure 2B) SEM showed that RBMSCs adhered to the surface of porous scaffolds. After 7 days of incubation, the cells showed an irregular shape in the pure scaffold group, while in the ECM–loaded scaffold group, cells on the scaffold surface became long and spindle–shaped with extended filopodia. (Figure 2C) The expression levels of osteogenic (RUNX2, BMP2) and vascularized genes (CD31, VEGF) were detected by RT–PCR at Day 0, 7 and 14. The results showed that the mRNA expression levels of a series of genes in RBMSCs were upregulated in the ECM–loaded scaffold groups. In particular, the gene expression levels in the RAOEC–ECM scaffold group were significantly increased compared to those in the other groups (*p* < 0.05).

### 3.3 Images of the surgical procedure and results

**FIGURE 3:**
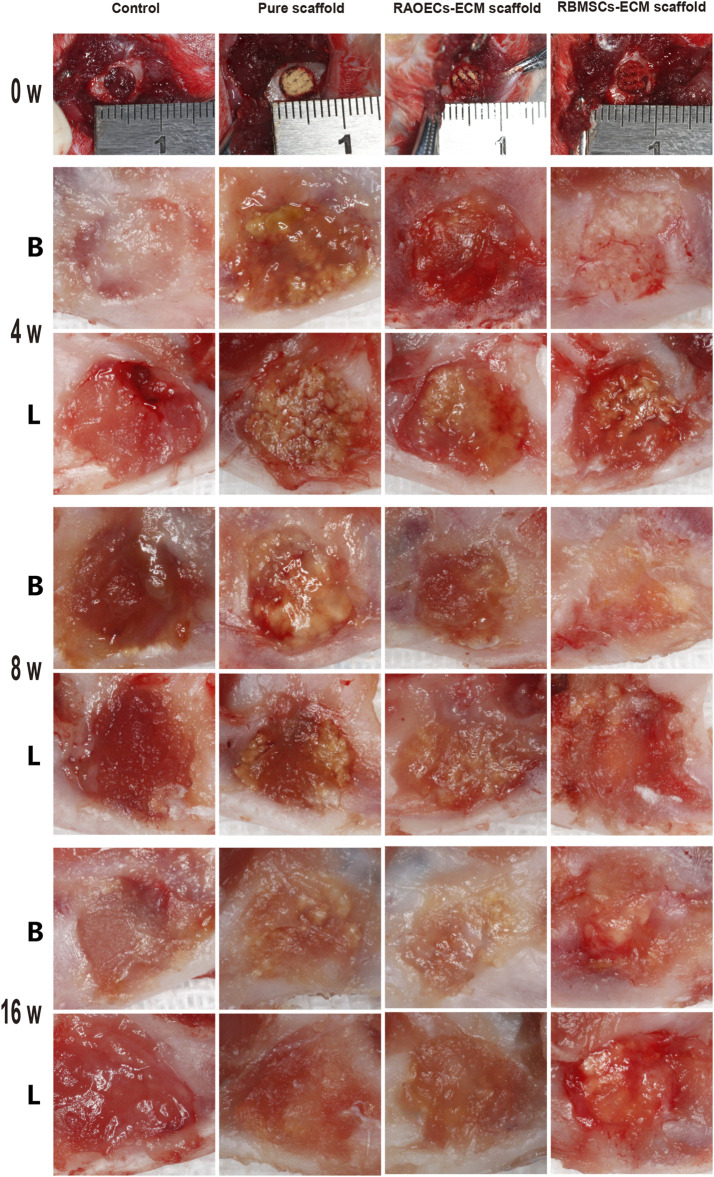
Photographs of the surgical procedure and postoperative healing. The structural integrity of all the scaffolds was maintained during surgery. At 4, 8 and 16 weeks after surgery, the defect sites of each group were observed from buccal (B) and lingual (L) angles.

As shown in Figure 3, during the surgical process, the prepared scaffolds fit the defects well and were easy to handle. After 4, 8 and 16 weeks, mandibular samples were harvested to characterize the status of defect closure. In the blank control group, a large amount of fibrous connective tissue was observed in the defect area, while in the scaffold–implanted groups, the scaffold materials were stably combined with the defects, and the material surface was covered by new fibrous connective tissue. The scaffold materials in the scaffold–implanted groups were partially degraded but still existed at 16 weeks after surgery. All defects healed uneventfully, with no evidence of wound dehiscence during healing, and no infection or necrosis was observed at the buccal and lingual sites.

### 3.4 Micro–CT analyses

**FIGURE 4:**
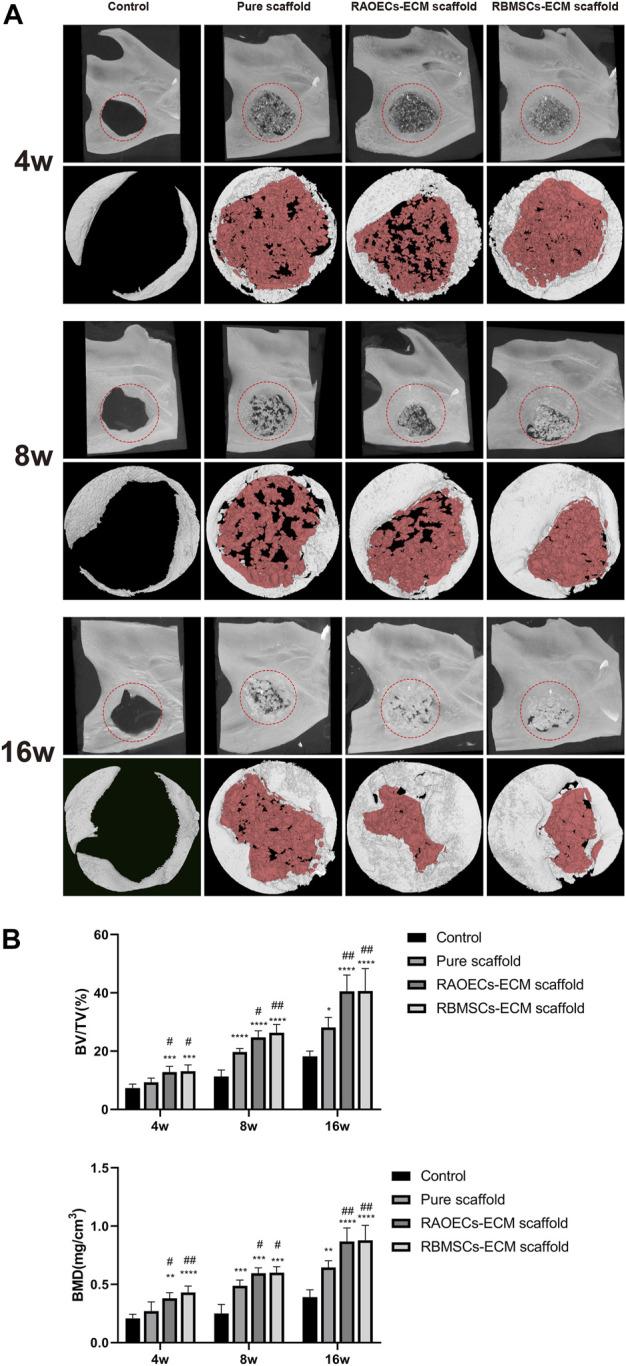
Micro–CT analysis of the control, pure scaffold, RAOECs–ECM scaffold and RBMSCs–ECM scaffold groups at 4, 8 and 16 weeks. **(A)** Three–dimensional images (top) and reconstruction images (bottom) are shown; white indicates new bone, and red represents residual scaffold materials. **(B)** Bone regeneration analysis of the ratio of the bone volume/tissue volume (BV/TV%) and bone mineral density (BMD, mg/cm^3^). (Significant effect compared to the control group: ∗*p* < 0.05, ∗∗*p* < 0.01, ∗∗∗*p* < 0.001, ∗∗∗∗*p* < 0.0001. Significant effect compared to pure scaffold: #*p* < 0.05, ##*p* < 0.01, *n* = 5.)

Micro–CT analyses were used to verify postoperative new bone formation and residual bone defect areas at different weeks (Figure 4). Representative 3D reconstruction images are shown in Figure 4A. New bone and scaffold materials are presented in white and red, respectively. At 4 weeks, new bone was identified around the defect margins in all groups. Scaffolds treated with ECM showed more newly formed bone tissues than the control and pure scaffold group. At 8 weeks, more new bone tissues grew into defects in all groups. Newly formed bone tissues surrounded the residual materials and narrowed the defect areas in the scaffold–implanted groups. In the control group, new bone tissues mainly appeared at the borderline of the bone defect, which was significantly lower than that in the scaffold–implanted groups. At 16 weeks, new bone tissues filling the defect areas were more significant in the ECM–loaded scaffold groups than in the other groups. Moreover, the materials of the scaffold groups loaded with ECM were partially degraded but still remained. Accordingly, quantitative analysis of the newly regenerated bone in the defects was detected by calculating BV/TV and BMD (Figure 4B). The pure scaffolds showed higher values than the control group. Moreover, the two kinds of ECM–loaded scaffolds had significantly higher values than the pure scaffold at all different time points (*p* < 0.05). In particular, there was no significant difference in either ECM–loaded scaffolds (*p* > 0.05). All of these results suggested that Gel/SA/58sBG scaffolds had a positive effect on promoting new bone regeneration during the defect repair period, and this effect could be strengthened when they were loaded with ECM from RAOECs or RBMSCs.

### 3.5 Analyses of neovascular formation

**FIGURE 5:**
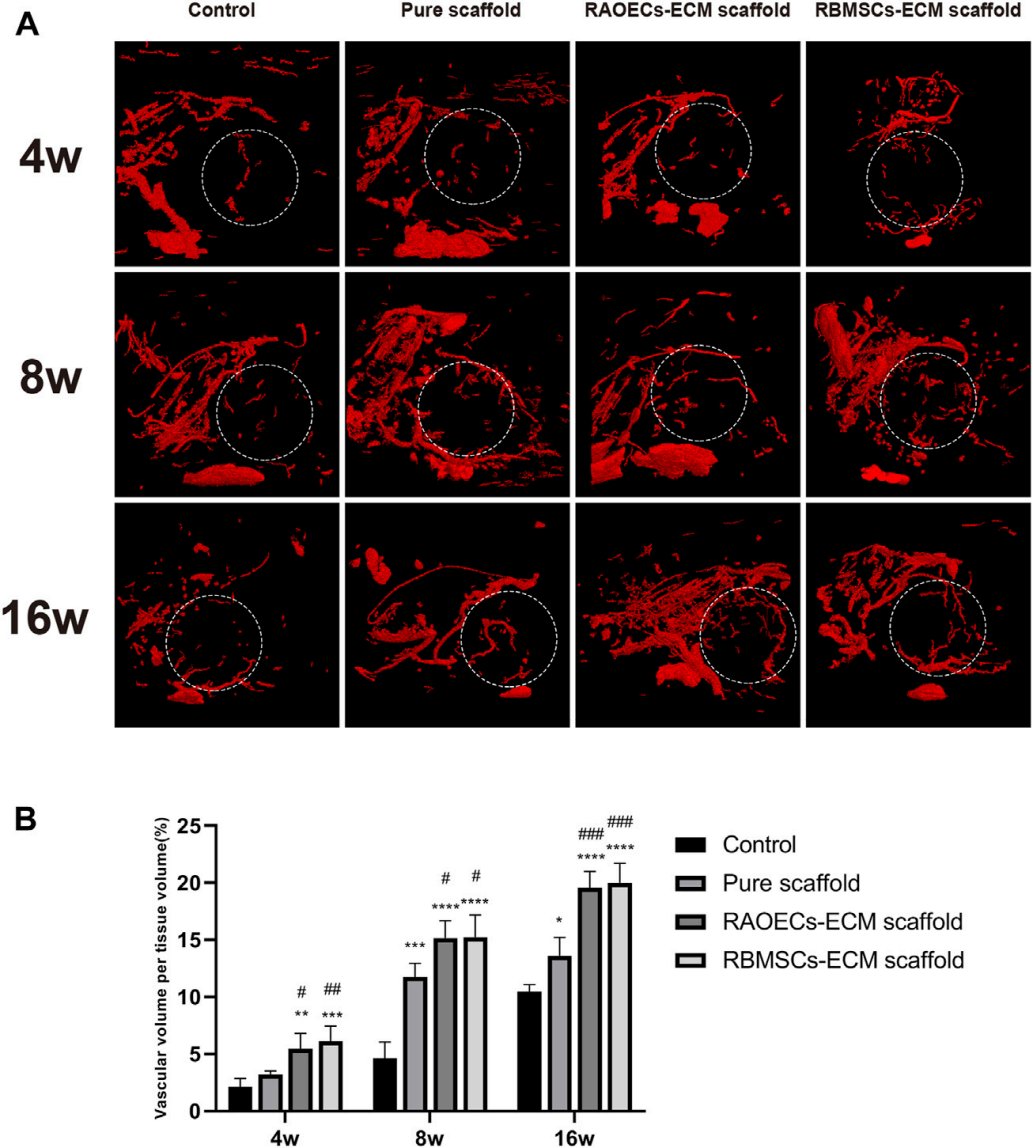
The vascular volume at different mandible defects was examined by Micro–CT at 4, 8, and 16 weeks after the operation. **(A)** 3D–reconstructed blood vessel images. **(B)** Quantitative analysis of vascular volume in the bone defect areas. (Significant effect compared to control group: ∗*p* < 0.05, ∗∗*p* < 0.01, ∗∗∗*p* < 0.001, ∗∗∗∗*p* < 0.0001. Significant effect compared to pure scaffold: #*p* < 0.05, ##*p* < 0.05, ###*p* < 0.001, *n* = 4.)

The blood vessel formation effect of each group was studied by Microfil perfusion and Micro–CT imaging after implantation (Figure 5). Images of microangiography revealed newly formed blood vessels distributed in the defect areas. Compared with the control group, the scaffold–implanted groups had more neovascularization, and the vascular network was denser. This phenomenon was more obvious with increasing time. Moreover, the vascular networks in both ECM–loaded scaffolds were abundant, and the newly formed blood vessels extended along the defect areas at 16 weeks. Both ECM–loaded scaffolds displayed more new blood vessels than the pure scaffold at each time point. Quantitative analyses of neovascular formation corresponded to the above results, which showed that both ECM–loaded scaffold groups had significantly higher new vessel volumes than the other groups (*p* < 0.05). These results indicated that ECM–loaded scaffolds could effectively promote neovascularization in the process of repairing defective bones.

### 3.6 Histological assessment

**FIGURE 6:**
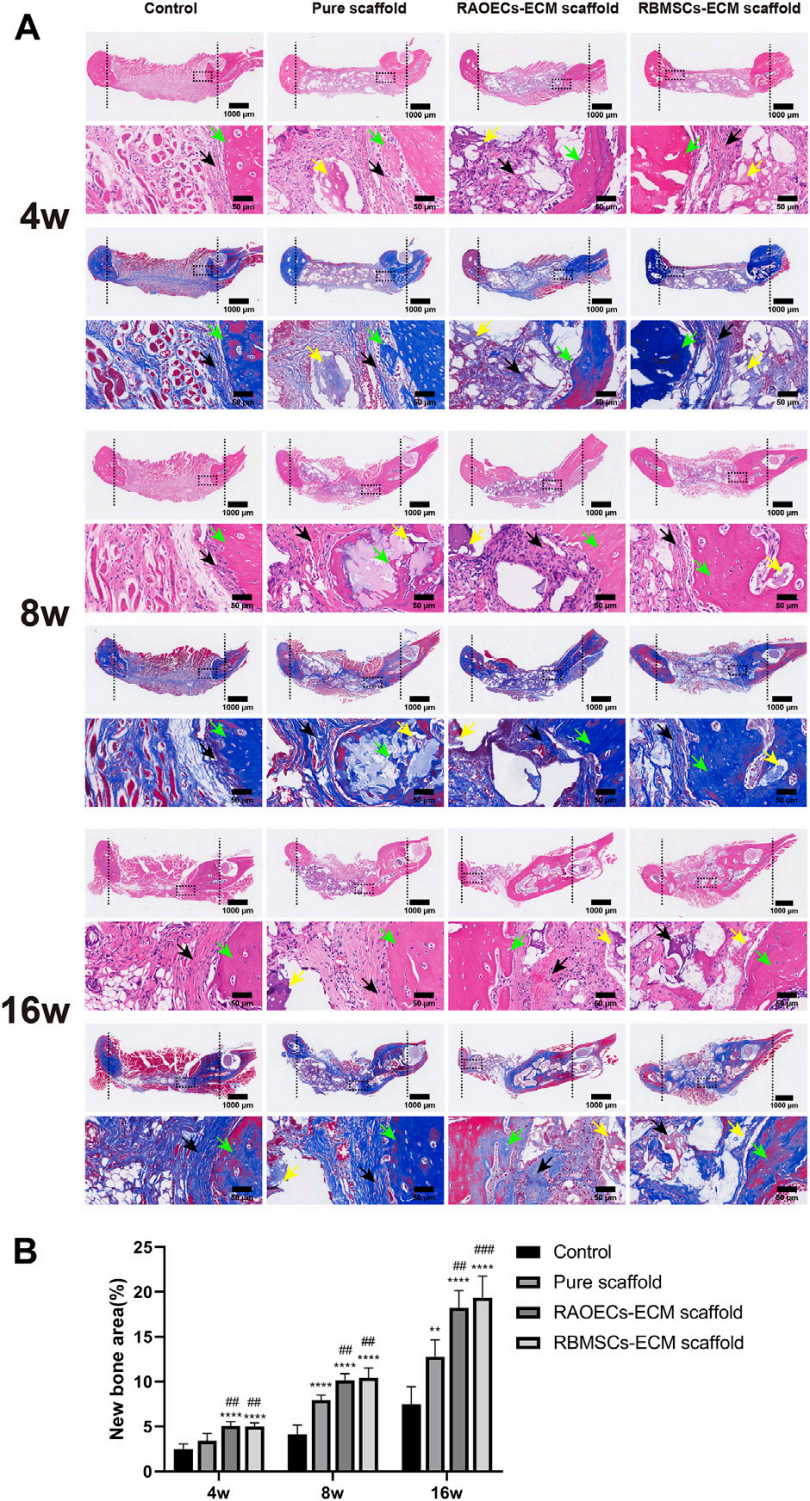
**(A)** H&E and MT staining of the cross–sections showed the histological morphology in the mandibular bone defects at 4, 8 and 16 weeks (black arrows: fibrous tissues, green arrows: new bone, yellow arrows: residual materials). **(B)** Quantitative histomorphometric analyses of new bone area. (Significant effect compared to control group: ∗∗*p* < 0.01, ∗∗∗∗*p* < 0.0001. Significant effect compared to pure scaffold: ##*p* < 0.01, ###*p* < 0.001, *n* = 5.)

**FIGURE 7:**
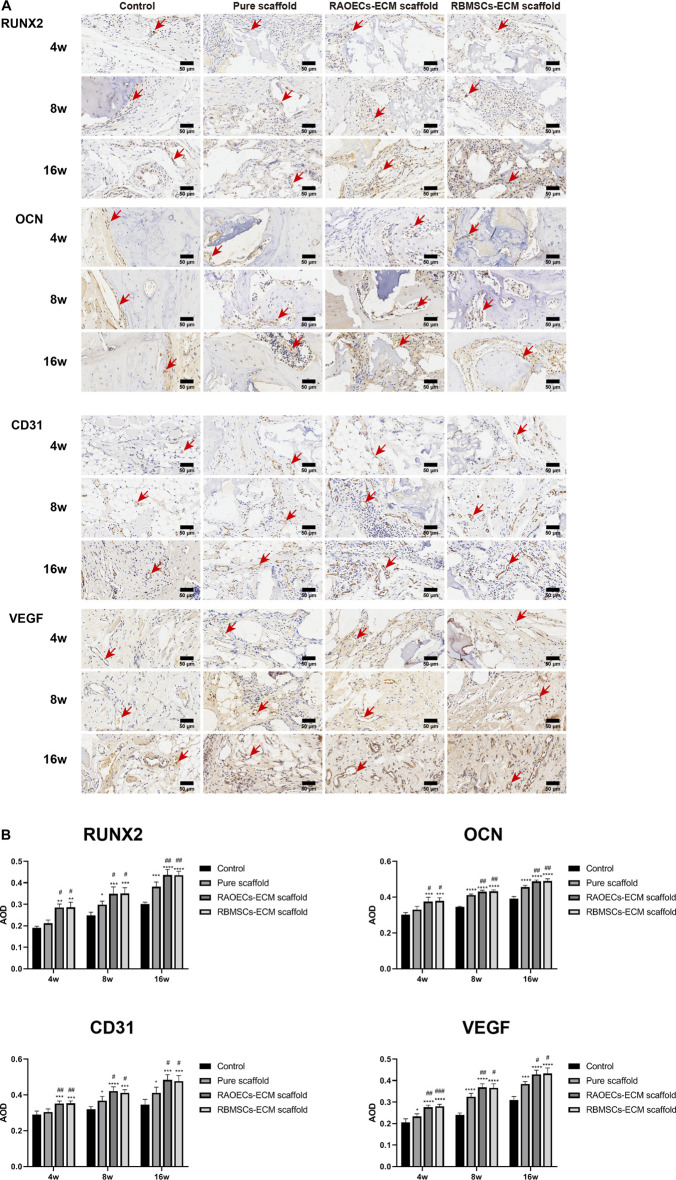
(Continued). **(A)** Images of immunohistochemical staining of RUNX2, OCN, CD31 and VEGF in bone defects. Bone regeneration activity was defined aspositive staining for RUNX2 and OCN. Blood vessel formation was indicated by positive staining for CD31 and VEGF. Positive staining for RUNX2, OCN, CD31 and VEGF was obvious in RAOECs and RBMSCs–ECM–loaded scaffolds, which had significant hyperchromatism compared with theother groups. **(B)** Quantitative analyses of the positive staining area. (Significant effect compared to control group: *p < 0.05, **p < 0.01, ***p < 0.001, ****p < 0.0001. Significant effect compared to pure scaffold: #*p* < 0.05, ##*p* < 0.01, ###*p* < 0.001, *n* = 4).

Histologic analysis performed with H&E and MT staining further revealed the condition of the bone defect at different time points. As shown in Figure 6, in the H&E staining, the newly formed bone sprouted from the margin of the defects in all groups at different weeks, as confirmed by the typical structure of sparse osteocytes embedded in lacunas and osteoblasts lining the outer edge of the bone tissue. In the control group, large amounts of loose fibrous tissues (black arrows) in the defect areas were observed at each time point, indicating poor bone regeneration capacity. In contrast, the groups implanted with scaffolds exhibited relatively reduced infiltrative growth of fibrous tissues and increased formation of dense bone tissue at the defect sites. Additionally, a significantly larger amount of new bone (green arrows) was observed in the ECM–loaded groups than in the pure scaffold group. Moreover, residual materials (yellow arrows) were also observed in the scaffold–implanted groups at 16 weeks, which were infiltrated by vast collagen tissues. Following MT staining, collagenous tissue and new bone were stained blue, while muscular tissues and mature lamellar bone were stained red. In the control group, limited new collagen and new bone formation were observed in the defect area at each time point, which was predominantly filled with loose fibrous tissue. However, the newly formed collagenous and bone tissue were more obvious in the scaffold–implanted groups. Especially at 16 weeks, mature lamellar bone and bridging trabeculae were generally most evident in ECM–loaded scaffolds, which was similar to the H&E staining results. Immunohistochemical staining of decalcified mandibles was performed to test the osteogenic markers RUNX2 and OCN and the angiogenic markers CD31 and VEGF. Positive staining in tissue slicing was dyed brown. The staining results showed a smaller positive area in the control group. The scaffold–implanted groups showed obvious positive areas for RUNX2 and OCN. Positive staining of RUNX2 and OCN accumulated in the tip of the newly formed collagen and bone tissue. Notably, both ECM–loaded scaffolds had significantly higher expression levels of RUNX2 and OCN than the pure scaffold group. Additionally, the most pronounced positive staining of CD31 and VEGF was also observed in the ECM–loaded scaffold groups (Figure 7A). The percentage of positive staining area of osteogenic and angiogenic markers suggested that the ECM–loaded scaffold groups were more significant than the other groups (Figure 7B). These results showed that ECM–loaded scaffolds can effectively promote the expression of osteogenic and angiogenic markers in the bone defect area, and the effect was better than those of the pure scaffold group and control group.

### 3.7 Biosafety assessment

**FIGURE 8:**
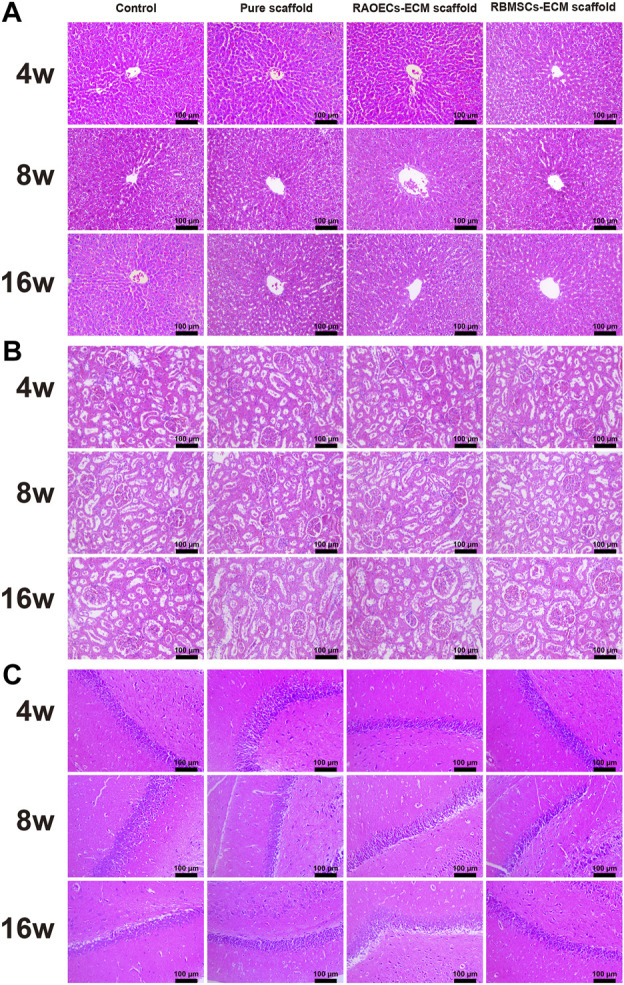
H&E staining of the liver **(A)**, kidney **(B)** and cerebrum **(C)** of rats in the control, pure scaffold, RAOECs–ECM scaffold and RBMSCs–ECM scaffold at 4, 8 and 16 weeks. No abnormalities were detected in any group.

Blood biochemical analysis data are shown in Table 2. Compared to the control group, no statistically significant changes were revealed in any parameters in the scaffold–implanted groups. Histopathologic examinations of the liver, kidney and cerebrum revealed normal architecture and indicated a lack of morphologic disturbances in all experimental animals (Figure 8).

**TABLE 2:** Differences in some biochemical indices among groups of rats implanted with ECM–loaded scaffolds, pure scaffold and control at different times after implantation.

A				
**Parameter**	**Control**	**Pure scaffold**	**RAOECs–ECM scaffold**	**RBMSCs–ECM scaffold**
TP (g/L)	76.24 ± 5.42	80.52 ± 6.47	71.80 ± 5.31	70.64 ± 8.52
ALB (g/L)	40.24 ± 1.97	41.94 ± 1.20	39.12 ± 1.29	40.98 ± 1.89
ALT (U/L)	79.78 ± 38.12	76.76 ± 23.87	78.90 ± 43.34	70.54 ± 15.83
AST (U/L)	308.74 ± 76.43	297.04 ± 18.53	228.82 ± 14.91	249.20 ± 66.47
Cr (µmol/L)	47.80 ± 7.85	57.40 ± 11.41	56.00 ± 10.56	64.20 ± 15.66
BUN (mmol/L)	6.76 ± 0.60	8.78 ± 1.51	8.16 ± 2.40	6.90 ± 1.66
**B**				
**Parameter**	**Control**	**Pure scaffold**	**RAOECs–ECM scaffold**	**RBMSCs–ECM scaffold**
TP (g/L)	70.26 ± 2.93	73.20 ± 3.23	71.32 ± 2.24	70.42 ± 4.54
ALB (g/L)	37.08 ± 3.20	38.68 ± 1.58	38.46 ± 0.68	36.62 ± 1.79
ALT (U/L)	59.64 ± 29.16	58.88 ± 20.36	59.40 ± 12.05	72.02 ± 13.71
AST (U/L)	221.02 ± 87.58	231.16 ± 56.50	166.02 ± 22.98	197.36 ± 55.01
Cr (µmol/L)	57.80 ± 14.75	63.20 ± 25.64	72.40 ± 22.82	65.80 ± 9.91
BUN (mmol/L)	8.02 ± 3.24	7.68 ± 0.89	9.80 ± 3.00	6.92 ± 0.54
**C**				
**Parameter**	**Control**	**Pure scaffold**	**RAOECs–ECM scaffold**	**RBMSCs–ECM scaffold**
TP (g/L)	71.80 ± 2.30	72.56 ± 5.80	69.60 ± 3.57	67.14 ± 5.06
ALB (g/L)	37.90 ± 1.80	38.66 ± 2.53	36.98 ± 3.01	36.84 ± 2.46
ALT (U/L)	74.04 ± 37.18	67.24 ± 29.93	68.12 ± 24.10	64.98 ± 23.71
AST (U/L)	235.12 ± 50.10	188.28 ± 39.22	199.96 ± 40.65	181.06 ± 23.29
Cr (µmol/L)	51.60 ± 21.82	81.80 ± 25.57	94.00 ± 24.32	63.00 ± 29.72
BUN (mmol/L)	7.46 ± 0.69	8.62 ± 2.03	9.26 ± 1.93	7.94 ± 2.15

Values expressed as the mean ± SD., Statistical analysis: One–way ANOVA, followed by Tukey’s multiple comparison test. A, B and C represent 4, 8 and 16 weeks after surgery, respectively. The indices of all groups showed no significant differences compared with those of the other groups at each time point (*p* > 0.05, *n* = 5).

## 4 Discussion

To date, one of the essential challenges in bone defect repair is the development of safe bone substitutes with good biological properties for the treatment of larger defects with complex shapes. For the application of bone tissue engineering, scaffolds display unique advantages, with pore structure providing transport channels for nutrients and metabolites for cell growth, facilitating cell proliferation and thus promoting bone regeneration. Recently, the development of 3D printing techniques has shown bright prospects, as this technology can be used to fabricate scaffolds with customized shapes and pore sizes as required (Lai et al., 2021). Hence, we prepared porous Gel/SA/58sBG scaffolds by 3D printing technology. Meanwhile, to modify the scaffolds, ECM was added to them to achieve rapid and sufficient angiogenesis and osteogenesis. ECM is an ideal alternative for regenerative medicine because most cells are strongly associated with the neighbouring ECM environment that supplies biophysical and biochemical signals for cell adhesion, migration, and differentiation (Lu et al., 2011; Choi et al., 2014). In bone tissue engineering research, synthetic scaffolds ornamented with native ECM may offer a cell microenvironment to improve the biological properties of scaffolds (Kim et al., 2018). In fact, decellularization of cells or tissue to generate ECM has been widely reported in tissue engineering. Among that, cell–derived ECM obtained by *in vitro* culturing has been identified as a feasible method for bone tissue engineering scaffold settings. Several approaches have been proposed to combine cells or other growth factors with scaffolds to achieve sufficient angiogenesis (Kanczler and Oreffo, 2008; Grellier et al., 2009; Kirkpatrick et al., 2011). Thus, in this study, prevascularization of Gel/SA/58sBG scaffolds was prepared by coating RAOECs–or RBMSCs–derived ECM, and RAOECs–ECM scaffold and RBMSCs–ECM scaffold were obtained. The prepared scaffolds with precisely controlled shapes are shown in Figure 1. All the scaffolds exhibited a rough surface with a microporous structure. The interconnected pores of scaffolds were potentially suitable for cell growth and nutrient delivery. ECM was homogeneously distributed in ECM–loaded scaffolds and showed fully loose granules. Many micropores could be seen on the surfaces of all scaffolds by SEM, with irregular filaments on the surfaces of ECM–loaded scaffolds.

Cell adhesion takes place in the early stage of tissue regeneration, which is essential to establish layers of cells for matrix maturation (Fragal et al., 2019). Proper cell adhesion is the premise of subsequent biological functions, such as cell migration and differentiation (Wu et al., 2020). As shown in Figure 2A, the Calcein AM staining results indicated that live RBMSCs seeded on the ECM–loaded scaffolds were more obvious than those in the control group, which may be attributed to the natural ECM coating, which provides a better niche and more binding sites for the cell interaction. Moreover, cells with long spindle shapes and extended filopodia could be seen on the surfaces of ECM–loaded scaffolds from the SEM view (Figure 2B). Most notably, RBMSCs were surrounded by some mineralized matter and spread out in ECM–loaded scaffold groups. For the pure scaffold, cells on the surface of the scaffold had a short spindle shape. This result indicated that scaffolds coated with ECM provide a rougher surface and a suitable environment for cell attachment and migration.

The osteogenic and angiogenic capacities of the ECM–loaded scaffolds were further verified *in vitro*. The eluates of the different scaffolds were collected after RBMSCs culture and subjected to RT–PCR (Figure 2C). The mRNA expression levels of osteogenic genes in ECM–loaded scaffolds, including RUNX2 and BMP2, were significantly upregulated compared with those in the pure scaffold and were regarded as the most representative markers for osteogenic differentiation and further contributed to bone regeneration *in vivo*. Moreover, bone tissue regeneration *in vivo* also derives directly from the differentiation of osteogenic stem or progenitor cells (Xu et al., 2021). In particular, among the three groups of scaffolds, RAOECs–ECM scaffold showed the highest expression of osteogenic genes *in vitro*. We inferred that the extracellular matrix of RAOECs has a significant influence on the differentiation of RBMSCs (Lozito Thomas et al., 2009; Saleh Fatima et al., 2011).

The increased expression levels of angiogenic genes in RT–PCR demonstrated that scaffolds loaded with RAOECs–derived ECM presented excellent angiogenic differentiation ability, followed by RBMSCs–ECM scaffold. Moreover, both CD31 and VEGF were evidently expressed at the center of the defects in both kinds of ECM–loaded scaffolds, which further proved that scaffolds decorated with RAOECs–or RBMSCs–derived ECM significantly facilitated angiogenesis. Moreover, 3D reconstruction images of the Microfil (contrast medium)–based angiographic technique for evaluating vascular morphology in Figure 5 also showed more vessel formation in both ECM–loaded scaffold groups. Cumulative evidence demonstrated that loading RAOECs–or RBMSCs–derived ECM in scaffolds can accelerate the formation of blood vessels, which facilitated new bone regeneration and, eventually, mature bone formation in the defect sites.

To test the osteogenic effect of implanted materials *in vivo*, it is usually necessary to set up a bone defect model, which must satisfy the requirement that the defect area be large enough to avoid complete self–healing without any treatment and thus prevent overestimation of spontaneous healing in a surgically created defect. The ideal state is to form new bone when only the bone repair material is present to verify the bone repair effect of the materials. A critical size defect (CSD) is the smallest bone defect that cannot completely heal itself in laboratory animals under natural conditions. At present, when the material is used to repair the mandibular defect in rats, a 5–mm–diameter perforating bone defect is usually created in the mandibular ascending ramus, which is considered the critical bone defect of the rat mandible (Zhang et al., 2016; Yu et al., 2018; Zhang et al., 2018). Therefore, the ECM–loaded scaffolds were implanted into critical–sized defects of the mandibles of rats to detect the bone repair potential in this study. After 4, 8 and 16 weeks of implantation, new bone ingrowth was detected and analysed by Micro–CT (Figure 4). The quantitative CT results illustrated that the repaired defects with ECM–loaded scaffolds had the following characteristics: higher bone volume fraction and bone density compared to both the pure scaffold and the blank control group. Histological analysis was used to provide a more detailed analysis of the bone response to the scaffolds with or without ECM, the results of which are shown in Figure 6. In the control and scaffold groups, H&E images showed that defects were filled with fibrous connective tissue with sparse new bone formation at the edge of the host bone. In contrast, in the ECM–loaded scaffold groups, residual materials surrounded by fibrous connective tissue could be observed in the defects, with a large amount of new bone formation at the edge. Moreover, RUNX2 expression was increased, and OCN staining was obviously deposited in the newly generated tissue in both ECM–loaded scaffold groups (Figure 7). RUNX2, an early osteogenic marker activating the development of stem cells into preosteoblasts, is the main regulator of osteoblast differentiation and acts as the determinant of bone remodelling and skeletal integrity (Ngo et al., 2020; Yang et al., 2021). OCN secreted by mature osteoblasts is a key marker in later–phase osteogenic differentiation (Quan et al., 2019). The obvious expression of RUNX2 and OCN also suggests that scaffolds combined with RAOECs–or RBMSCs–derived ECM can accelerate bone formation in the early and later stages of bone defect healing. In Masson’s trichrome staining, blue staining is indicative of collagen fibrous tissue and osteoid. This staining could serve as a good indication for bone tissue formation because of the enrichment of collagen in the bone matrix (Zhao et al., 2018). In the control and pure scaffold groups, limited collagen and new bone deposition were observed in the defect area compared to the other groups at each time point. Notably, a large amount of collagen and new bone components were detected in the defect sites implanted with the ECM–loaded scaffolds, indicating the improved osteogenic ability of the ECM. These results indicated that the presence of ECM–loaded scaffolds provides a bone–like environment for bone mineralization with the contribution of functional ECM molecules.

The results *in vitro* and *vivo* show that ECM–loaded scaffolds have better bone regeneration effect than the pure one. Possible reasons are as follows: 1) RAOECs–or RBMSCs–ECM contains several kinds of bioactive growth factors and various functional proteins that contribute to cell recruitment, which is the first step for cells and materials interaction, and the better cell recruitment is more beneficial to subsequent cell proliferation and tissue regeneration. 2) In addition, when RAOECs–or RBMSCs–ECM loaded on scaffolds, microporosity of the ECM provided some advantages, such as more binding sites for protein combination, which is an effective approach to improve tissue repair and regeneration. At the meantime, the surface and interior of scaffolds are coated by ECM, which imitate the biological macro and microenvironment to promote cell migration and invasion into the scaffolds to realize self-assembly of cells. 3) ECM could regulate cell differentiation by concerning mechanical and biochemical signals, which provide cellular support to modulate the biological and physical cues and further achieve bone regeneration.

The biosafety of a material should be considered when it is directly implanted into the body. Biosafety assessments generally refer to a series of evaluation standards for medical biomaterials and devices. When the scaffolds are implanted in the defects, they can wear out and separate free particles in the process of mandible movement, which may cause embolism or inflammation (Koff Matthew et al., 2019; Qiu et al., 2020). Normal physiological activities can be affected by this phenomenon. Therefore, postimplantation local reactions and systemic toxicity tests were employed in our study. The application of the local reaction test after implantation in the bone defect model is mainly achieved by means of gross observation and histological staining of the implanted area. Based on our observations, all the experimental animals could eat on their own within 3 days after surgery, the defect areas healed well in the scaffold–implanted groups after surgery, and no adverse response related to inflammation in the defect areas was aggravated. This response is regarded as the bioglass of scaffolds exhibiting chemotactic activity for inflammatory cells (Zhang et al., 2020). The biosafety performance of the scaffolds was investigated in the mandibular defects of rats over 3 months. The scaffold–implanted areas showed no infection or necrosis at either site (Figure 3). In addition to observing the biocompatibility of the material with surrounding tissues, *in vivo* toxicity tests are also needed to identify any toxic damage caused by the material itself or its degradation products to vital organs. Lai et al. (2019) evaluated the biosafety of materials *in vivo* by detecting changes in related indicators of liver and kidney function after the implantation of materials into bone defects. Jia et al. (2021) evaluated the toxicity of implant materials for bone defects *in vivo* by detecting pathological changes in the liver, kidney and other important organs. In this study, the blood biochemical indices of the scaffold–implanted groups showed no significant differences compared to the control group (Table 2). The H&E staining of vital organs in the scaffold groups showed no obvious abnormalities (Figure 8). All of the *in vitro* and *in vivo* results demonstrated that the 3D–printed scaffolds presented admirable biosafety, indicating that the implanted scaffolds circumvented the side effect of movement of the mandible and could still perform certain functions under specific size defects in place of the defective bone. This also indicates that the scaffolds were qualified for treating the mandibular defects caused by clinical diseases.

In summary, although these ECM–loaded scaffolds showed acceptable osteogenic effects, targeted improvements of scaffolds for repairing large animal models to reveal craniomaxillofacial bone healing more accurately could be our next work, which would include combining the different ECMs in various proportions to enhance the osteogenic effect.

## 5 Conclusion

The results of our study show that individualized scaffolds are easily fabricated using 3D printing technology and that RAOECs–or RBMSCs–derived ECM is an ideal factor for accelerating osteogenesis and angiogenesis. *In situ* bone defect repair with of RBMSCs–ECM scaffold or RAOECs–ECM scaffold clearly promoted bone regeneration and neovascularization. Moreover, the scaffolds showed good biosafety *in vitro* and *in vivo*. Thus, the combination of Gel/SA/58sBG scaffolds with RAOECs–or RBMSCs–derived ECM may provide an effective strategy for treating bone defects. However, due to the complexity of the RAOECs–derived ECM and RBMSCs–derived ECM components and functions, it is hard to identify the specific mechanism of the osteogenesis. In addition, is there a synergistic osteogenesis effect when RAOECs–derived ECM and RBMSCs–derived ECM are mixed in a certain proportion? These two still needed further research in the future.

## Data Availability

The datasets presented in this study can be found in online repositories. The names of the repository/repositories and accession number(s) can be found in the article/supplementary material.
